# Clinical variations in Parkinson’s disease patients with or without REM sleep behaviour disorder: a meta-analysis

**DOI:** 10.1038/srep40779

**Published:** 2017-01-16

**Authors:** Ruo-lin Zhu, Cheng-juan Xie, Pan-pan Hu, Kai Wang

**Affiliations:** 1 Department of Neurology, The First Affiliated Hospital of Anhui Medical University, Hefei, China; 2 Department of Medical Psychology, Anhui Medical University, Hefei, China; 3 Collaborative Innovation Centre of Neuropsychiatric Disorders and Mental Health, Anhui Province, China

## Abstract

This study aimed to evaluate the clinical variations in patients with Parkinson’s disease (PD) with (PDRBD) or without REM sleep behaviour disorder (RBD) (Non-RBD), and PDRBD patients were classified into Confirmed-RBD (definite diagnosis with polysomnography, PSG) and Probable-RBD (without PSG re-confirmation). The clinical difference between the groups of patients was measured as an odds ratio (OR) or standardized mean difference (SMD, Cohen d). A total of 31 articles with data from 5,785 participants were obtained for our analysis. Overall, the occurrence of Confirmed-RBD was more frequent in male patients (OR = 1.25; p = 0.038), elderly patients (SMD = 0.25; p = 0.000), and patients with longer disease duration (SMD = 0.30; p = 0.000), increased Hoehn-Yahr scale (SMD = 0.30; p = 0.000), and higher UPDRS-III score (SMD = 0.38; p = 0.002). On the other hand, the frequency of Probable-RBD was increased with disease duration (SMD = 0.29; p = 0.000), Hoehn-Yahr scale (SMD = 0.30; p = 0.000), and UPDRS-III score (SMD = 0.26; p = 0.001). Our study indicate that PDRBD patients may have different clinical features compared to patients with Non-RBD.

REM sleep behaviour disorder (RBD), a parasomnia that is characterized by a loss of normal skeletal muscle atonia during REM sleep and elaborate motor activity associated with oneiric content, is one of the most common non-motor symptoms of Parkinson’s disease (PD)^1^,^2^. Epidemiological investigation demonstrated that RBD, which has a prevalence of <1% in the general population^3^,^4^, is a common disorder that occurs in 15–75% of patients with PD, especially males over 50 years of age^5^,^6^,^7^,^8^. RBD frequently leads to serious injuries to PD patients, e.g., falling out of bed, and patients can harm their sleeping partner while dreaming^1^,^9^.

RBD is considered a heralding disorder or early marker of α-synucleinopathies, including dementia with Lewy bodies (DLB), multiple system atrophy (MSA), and Parkinson’s disease (PD)^10^,^11^,^12^. A longitudinal cohort study revealed that RBD appears several years or even decades before the onset of the motor symptoms of PD^13^. Furthermore, 38% of patients developed a parkinsonian syndrome approximately 3.7 years after an initial diagnosis of idiopathic RBD^10^.

Current research suggests that neuronal degeneration of brainstem nuclei, including the pontine tegmental area and medulla, is the pathophysiological cornerstone of RBD^14^. However, there are still a remarkable number of PD patients who do not display RBD symptoms, even with long disease duration. Thus, there are likely additional factors involved in the occurrence of PD-related RBD (PDRBD) other than neuronal degeneration.

Numerous studies are available on the prevalence, clinical characteristics and disease staging of RBD. However, systemic meta-analyses in large samples for comparing the clinical variations between patients with PDRBD and those with PD alone (Non-RBD) are scarce. Therefore, the primary aims of our study were to compare the clinical characteristics of PDRBD and Non-RBD. This study was conducted with data originating from multiple studies with an overall moderate quality.

## Methods

### Data sources and search strategy

The study protocol was approved by the Clinical Ethics Committee of The First Affiliated Hospital of Anhui Medical University. In accordance with the Preferred Reporting Items for Systematic Reviews and Meta-Analyses (PRISMA) Statement^15^, we undertook a systematic search of PubMed and Embase databases for studies published between January 2000 and August 2016 with a language limitation of English only. The search terms “rapid eye movement”, “rem”, “rem sleep behavior disorder”, or “rapid eye movement sleep behavior disorder” were combined using the Boolean logical operator AND with studies identified by the terms “parkinson”, “parkinson’s”, “parkinson disease”, “parkinson’s disease”, “parkinsonian”, “parkinsonian disease” or “parkinsonian disorders”. The references in the primary selected articles, relevant reviews and meta-analyses were also examined to identify additional relevant studies.

### Data extraction and quality assessment

Prospective or retrospective case-control studies that compared PD patients with and without RBD were eligible for inclusion in our meta-analysis. The papers were initially screened on the basis of their titles and/or abstracts by two reviewers (Ruo-lin Zhu and Cheng-juan Xie). For inclusion in the meta-analysis, a study had to meet the following criteria: (1) the original data were published; (2) the diagnosis of PD was confirmed with the U.K. Parkinson’s Disease Society Brain Bank criteria or other criteria; (3) the PD incidence data were classified into groups of PDRBD and Non-RBD. Notably, in this meta-analysis, a definition of Confirmed-RBD was made when RBD patients met the criteria described in the International Classification of Sleep Disorders (ICSD), in which polysomnography (PSG) is mandatory; otherwise, the Probable-RBD was defined in RBD patients who were diagnosed based on interview or questionnaires. (4) the study described the number of participants and PD cases, ratio or number of male/female subjects, age, onset age of PD, disease duration, Hoehn-Yahr staging scale for disease activity, and the Unified Parkinson’s Disease Rating Scale part III (UPDRS-III) score for motor symptom severity. Studies with inadequate data on the diagnosis of PD and/or RBD and those focused on pathogenic mechanisms were excluded. Articles that included fewer than 20 patients and studies based on animal research were also excluded.

Bias risk and applicability were critically evaluated based on a quantitative 5-point Jadad scale^16^. The Jadad scale focuses on 4 critical items, including randomisation (maximum of 2 points), blinding (maximum of 2 points) and an account of all patients (maximum of 1 point). The Jadad scores were determined by two reviewers (Ruo-lin Zhu and Cheng-juan Xie). Scoring disagreements were determined by an arbitrator (Kai Wang).

### Statistical analysis

The meta-analysis of dichotomous or continuous variables was conducted using the Mantel-Haenszel method with a fixed- or random-effects model (based on the heterogeneity analysis). Data were combined and expressed as odds ratios (ORs) or standardized mean differences (SMDs) for dichotomous and continuous variables, respectively, with 95% confidence intervals (95% CI).

Forest plots were presented with a measure of inconsistency across the trials (the Cochrane Q statistic was calculated from Chi^2^ and the I^2^ statistic) and a test for overall effects (Z). The statistical heterogeneity between studies was assessed with the Cochran’s Q-test and I^2^-values. If the Cochran’s Q test had a p value < 0.05, an I^2^ value over 75% was defined as high inconsistency, an I^2^ above 50% was defined as moderate heterogeneity, and an I^2^ below 25% was defined as low levels of inconsistency amongst the included studies^17^,^18^,^19^.

Funnel plots were produced to detect potential publication bias. All statistical tests and the construction of forest plots were performed using Stata/SE version 13.1 (StataCorp, College Station, TX, USA). The significance level was set at p < 0.05.

## Results

### Study identification and methodological quality

**Figure 1:**
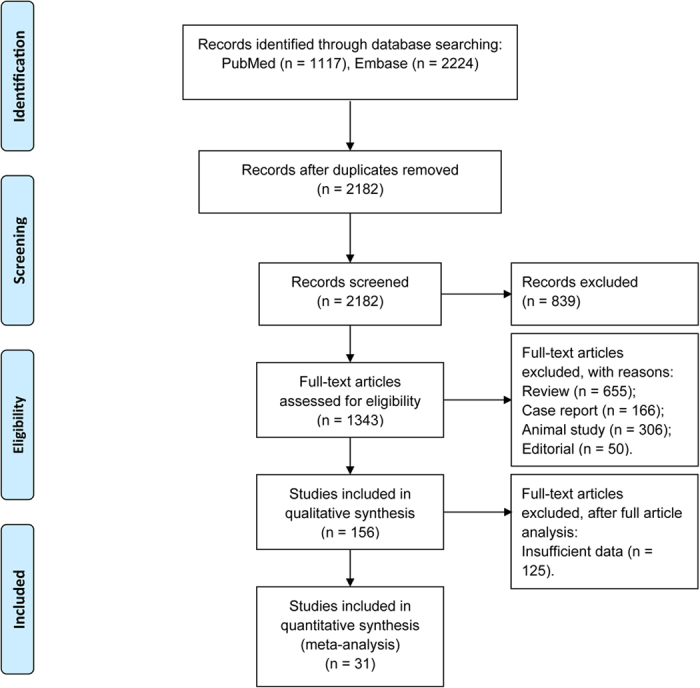
Study flow chart.

The initial search strategy yielded 3,341 potentially relevant studies from the PubMed (n = 1,117) and Embase (n = 2,224) databases. After 1,159 duplicates were removed, the titles and/or abstracts of 2,182 citations were reviewed, and 839 studies were excluded. The remaining 1,343 full-text articles were retrieved for further evaluation. Another 1,187 studies were excluded because they were reviews (n = 655), case reports (n = 166), animal studies (n = 306) or editorials (n = 50). Ultimately, 31 articles^1^,^2^,^7^,^8^,^20^,^21^,^22^,^23^,^24^,^25^,^26^,^27^,^28^,^29^,^30^,^31^,^32^,^33^,^34^,^35^,^36^,^37^,^38^,^39^,^40^,^41^,^42^,^43^,^44^,^45^,^46^ were included in our analysis (Fig. 1). The diagnosis of Confirmed-RBD and Probable-RBD were defined in 19 and 12 studies respectively (Table 1). No disagreements on study selection occurred between the reviewers.

The studies used for in our meta-analysis consisted of 7,380 randomised subjects, which ultimately encompassed 5,815 participants (PDRBD, n = 2,319, mean group size = 74.8; and Non-RBD, n = 3,496, mean group size n = 112.8) (Table 1). Fourteen studies were performed in Asia, 11 in Europe, 5 in North America, and 1 in Australia. The mean age across all subjects ranged from 54.1 to 76.5 years, and approximately 57.6% of patients were male. The onset age of PD ranged from 51.0 to 65.5 years. The mean disease duration of all participants ranged from 4.3–15.3 years. The mean Hoehn-Yahr scale and UPDRS-III score ranged from 0.9 to 3.2 and 0.23 to 190.97, respectively.

PDRBD patients were further classified into subgroups for two studies: clinical or subclinical RBD in the study conducted by Nomura *et al*.^36^ and PD-RBD with and without hallucinations in the study conducted by Sinforiani *et al*.^40^. The subgroup data were included independently in the meta-analysis for the variables of sex, age, disease duration, Hoehn-Yahr scale, and UPDRS-III score. The methodological quality was appraised for all studies included in the meta-analysis. Overall, the included studies were of moderate methodological quality according to the Jadad scale (Table 1).

### Summary of effects

**Figure 2:**
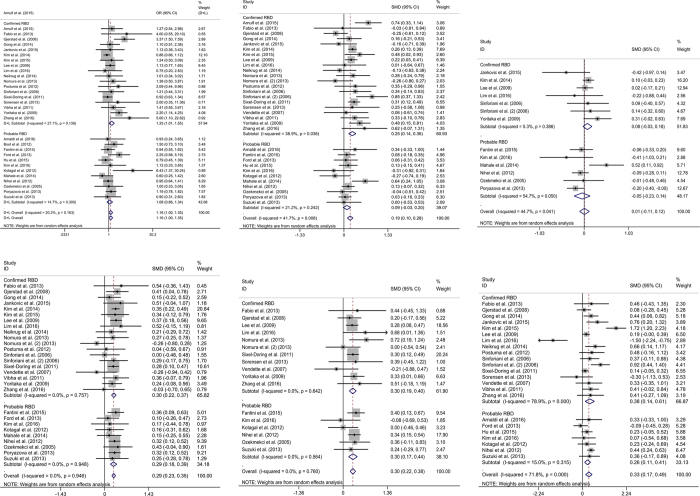
Forrest plot of the odds ratios (ORs) and standardised mean differences (SMD, Cohen d) for the clinical variations between PD patients with and without RBD (PDRBD and Non-RBD, respectively). If the Cochran’s Q test had a p value < 0.05, the I^2^ over 75%, above 50% and below 25% were defined as high, moderate and low inconsistency, respectively. (**A**) Gender, (**B**) patient age, (**C**) onset age of Parkinson’s disease, (**D**) disease duration, (**E**) Hoehn-Yahr staging scale, and (**F**) Unified Parkinson’s Disease Rating Scale part III (UPDRS-III) score.

**Figure 3:**
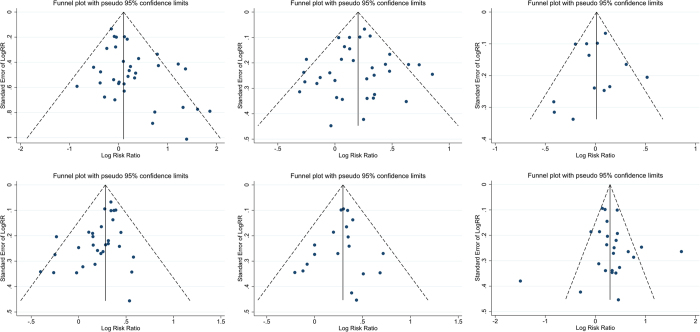
Funnel plot with pseudo 95% confidence limits for assessment of publication bias. (**A**) Gender, (**B**) patient age, (**C**) onset age of Parkinson’s disease, (**D**) disease duration, (**E**) Hoehn-Yahr staging scale, and (**F**) Unified Parkinson’s Disease Rating Scale part III (UPDRS-III) score.

With regard to sex, a total of 5,685 PD patients, including 2,252 PDRBD patients and 3,433 Non-RBD patients, from 30 studies were analysed. The study by Vendette *et al*.^43^ was not included because the gender ratio or number was not reported. The proportion of male patients was 57.8% (males in PDRBD, n = 1,298 and males in Non-RBD, n = 1,988). High-quality evidence that showed male patients had an increased risk of RBD. Overall, male patients with PD were more likely to develop Confirmed-RBD than female patients (OR = 1.25; 95% CI 1.01–1.55; p = 0.038); on the other hand, our meta-analysis showed no significant impact of gender on the occurrence of Probable-RBD in PD patients (OR = 1.08; 95% CI 0.86–1.34; p = 0.516). There was moderate heterogeneity across studies for analysis of Confirmed-RBD (I^2^ = 27.1%; p = 0.139) and no heterogeneity for Probable-RBD (I^2^ = 14.7%; p = 0.300) (Fig. 2A). The funnel plot did not suggest substantial asymmetry (Fig. 3A).

With regard to age, a total of 5,134 PD patients, including 2,292 PDRBD patients and 2,842 Non-RBD patients, from 30 studies were analysed. The study by Boot *et al*.^22^ was not included because the age of patients was demonstrated as a median (25^th^ percentile, 75^th^ percentile). Overall, elderly patients with PD were more likely to develop Confirmed-RBD than younger PD patients (SMD = 0.25; 95% CI 0.14–0.36; p = 0.000); and no significant effect of age on the occurrence of Probable-RBD (SMD = 0.09; 95% CI −0.02–0.24; p = 0.129). There was low heterogeneity across studies for analysis of Confirmed-RBD (I^2^ = 38.9%; p = 0.036) and no heterogeneity for Probable-RBD (I^2^ = 21.2%; p = 0.242) (Fig. 2B). The funnel plot did not show evidence of asymmetry (Fig. 3B).

The onset age of PD was reported in 12 studies, which included a total of 3,143 patients (1,448 PDRBD and 1,695 Non-RBD). The onset age of PD was 51.0 to 63.6 years for PDRBD patients and 48.6 to 65.5 years for Non-RBD patients. The meta-analysis showed no significant effect of onset age on the occurrence of Confirmed-RBD in PD patients (SMD = 0.08; 95% CI −0.03–0.18; p = 0.154); similarly, no significant effect of onset age on the occurrence of Probable-RBD (SMD = −0.05; 95% CI −0.23–0.14; p = 0.628). The inconsistency amongst the included studies was low (I^2^ = 5.3%; p = 0.386) and moderate (I^2^ = 54.7%; p = 0.050) respectively (Fig. 2C). No clear evidence of asymmetry was demonstrated in the funnel plot (Fig. 3C).

Twenty-eight studies reported a difference in disease duration between PDRBD and Non-RBD patients. A total of 2,623 PDRBD patients (mean disease duration from 4.8 to 15.3 years) and 2,111 Non-RBD patients (mean disease duration from 4.3 to 13.9 years) were included in the meta-analysis. A longer disease duration increased the risk of both of Confirmed-RBD and Probable-RBD in PD patients (SMD = 0.30; 95% CI 0.22–0.37; p = 0.000 and SMD = 0.29; 95% CI 0.19–0.39; p = 0.000). There was no significant inconsistency across the studies (I^2^ = 0.0%; p = 0.757 and 0.0%; p = 0.948 respectively) (Fig. 2D). The funnel plot did not suggest asymmetry (Fig. 3D).

Sixteen studies reported a difference in the Hoehn-Yahr score between PDRBD (n = 986) and Non-RBD patients (n = 1,496). The combined effect size was low but statistically significant for both of Confirmed-RBD and Probable-RBD (SMD = 0.30; 95% CI 0.19–0.40; p = 0.000 and SMD = 0.30; 95% CI 0.19–0.44; p = 0.000). There was no inconsistency across the studies (I^2^ = 0.0%; p = 0.642 and I^2^ = 0.0%; p = 0.564 respectively) (Fig. 2E), and the funnel plot did not suggest asymmetry (Fig. 3E).

Twenty-two studies described the UPDRS-III score for PDRBD patients (n = 1,162; mean score 1.19–38.7) and Non-RBD patients (n = 1,836; mean score 1.1–30.7). The UPDRS-III score was higher in Confirmed-RBD and Probable-RBD patients compared with Non-RBD patients (SMD = 0.38; 95% CI 0.14–0.61; p = 0.002 and SMD = 0.26; 95% CI 0.11–0.41; p = 0.001). True heterogeneity across studies was high for Confirmed-RBD (I^2^ = 78.9%; p = 0.0) and none for Probable-RBD (I^2^ = 15.0%; p = 0.315) (Fig. 2F). Two conspicuous studies^8^,^32^ that were out of range were revealed in the funnel plot (Fig. 2F). Removing these studies yielded a significant effect of the UPDRS-III score on the occurrence of RBD (SMD = 0.29; 95% CI 0.20–0.38; p = 0.000); however, heterogeneity was not observed across the studies (I^2^ = 17.7%; p = 0.221). Notably, UPDRS-III test performed on the state of “on”, “off” and unknown was reported in 3^8^,^40^,^43^, 4^8^,^30^,^40^,^44^ and 17^1^,^7^,^20^,^23^,^24^,^25^,^26^,^27^,^28^,^31^,^32^,^34^,^35^,^39^,^41^,^42^,^46^ of the original studies respectively, which is inappropriate for the further sub-group analysis in this meta-analysis. Therefore, the findings concerning the connection between UPDRS-III score and the occurrence of RBD should be interpreted more cautiously.

## Discussion

In the last decade, numerous studies evaluated the clinical factors associated with the occurrence of RBD in PD patients. However, the results are varied due to the diverse criteria for a diagnosis of RBD, different data sources and insufficient sample sizes in most studies. Thus, the relationship between RBD and PD remains unclear.

To summarize these previous studies and obtain a more comprehensive conclusion, we performed a meta-analysis of 33 studies with a total of 5,919 subjects who were diagnosed with PD, including 2,411 PDRBD patients. To our knowledge, this is the first meta-analysis that systematically estimated the clinical factors related to RBD in PD patients. Our findings demonstrated that Confirmed-RBD patients showed the following characteristics compared to PD patients with normal REM sleep behaviour: male, older, longer disease duration, higher disease activity (Hoehn-Yahr scale) and higher motor examination (UPDRS-III) score; while the Probable-RBD patients demonstrated the different clinical features compared to RBD patients in disease duration, disease activity and motor examination.

Previous studies suggested that the incidence of PD is greater in men than women due to the neuroprotective effects of oestrogen via stimulation of dopamine neurotransmission and greater levodopa bioavailability^47^,^48^ and/or the variant gene expression patterns in dopamine (DA) neurons in the substantia nigra pars compacta (SNc) between males and females^49^. Similar to previous reports, our meta-analysis demonstrated that male PD patients had a higher risk for RBD.

Evidence suggests that the pathophysiology of sleep disturbances in PD includes degenerative changes and structural lesions in brainstem areas, particularly the dorsal midbrain and pons, that are related to sleep-wake activity and sleep regulation^14^. With regards to clinical features, RBD was reported to be associated with older age, longer PD duration, higher disease severity expressed by Hoehn-Yahr scale, higher UPDRS motor and non-motor symptom scores, more severe motor fluctuation, and higher levodopa dosage for antiparkinsonian treatment, but there was no difference in onset age in PD patients when compared with Non-RBD patients^8^,^41^,^50^,^51^. These findings suggest that RBD in PD represents a more severe neurodegenerative process when compared with non-RBD.

At the time, our meta-analysis was conducted using all of the available literature sources that compared the clinical profiles of PD patients with and without RBD. Therefore, we consider these results to be comprehensive and valid. However, some limitations in our analysis and in the individual studies should be considered when interpreting our findings.

Previous studies revealed that antidepressants can produce dream-enactment behavior, reduce REM sleep atonia, and finally precipitate or aggravate RBD^22^,^52^,^53^. Even though the details for the mechanisms are unclear, the treatment of antidepressants especially selective serotonin reuptake inhibitors and serotonin-norepinephrine reuptake inhibitors have been implicated in the occurrence of RBD in patients with PD^22^. However, in this meta-analysis, we cannot distinguish primary RBD from RBD linked to antidepressants since the data were insufficient in the original articles. Besides, in several of the included studies^1^,^2^,^20^,^22^,^24^,^25^,^28^,^29^,^30^,^31^,^33^,^36^,^37^,^38^,^40^,^44^,^45^, RBD was diagnosed based on a questionnaire or clinical manifestations but not confirmed with PSG. PSG can detect muscle activation patterns during REM sleep, including aberrant chin muscle tone, limb jerking, and hyperkinetic or violent behaviour; however, this technique is not part of the mandatory criteria for a clinical diagnosis^2^,^54^. Regardless, PSG is considered the “gold standard” because it provides objective parameters of primary sleep behaviour in PD patients^55^. Thus, a lack of PSG confirmation for an RBD diagnosis might have led to a biased prevalence of RBD in PD patients in our meta-analysis.

Heterogeneity amongst the individual studies is inevitable in meta-analyses because of variability in design characteristics and the relatively poor quality of reporting in primary studies. In this study, several high-quality studies were removed due to data that were deemed inappropriate for our meta-analysis, i.e., data that would increase the heterogeneity and weaken the stringency of the meta-analysis. The deficiencies in the data prevented the inclusion of clinical symptoms (wearing-off/fluctuations, dyskinesia, hallucinations, restless legs syndrome, hypertension, or constipation), psychological assessment parameters (Beck Depression Inventory, BDI and Mini Mental State Examination, MMSE), and treatment (e.g., Levodopa dosage and course duration) in the meta-analysis.

In summary, our data suggest that the clinical characteristics between PD patients with and without RBD are distinct, indicating that PDRBD might represent a variant pattern of neurodegeneration in PD patients. However, the limitations of our study require a cautious interpretation of the identified risk factors for RBD in PD patients. Additional investigations with a more comprehensive design are needed to determine the distinct features of PDRBD patients.

## Additional Information

**How to cite this article**: Zhu, R.-l. *et al*. Clinical variations in Parkinson’s disease patients with or without REM sleep behaviour disorder: a meta-analysis. *Sci. Rep.* **7**, 40779; doi: 10.1038/srep40779 (2017).

**Publisher's note:** Springer Nature remains neutral with regard to jurisdictional claims in published maps and institutional affiliations.

## Figures and Tables

**Table 1:** Demographic and clinical characteristics of patients with Parkinson’s disease in the included studies.

Year	Author	Source	Diagnostic criteria for PD; Disease Characteristics	Diagnosis tool for RBD	Participants (n)	Age (mean ± SD, year)	Gender (M/F)	Jadad score
**Confirmed RBD**								
2015	Arnulf *et al*.	France	PDSBB	ICSD	114	RBD: 62.8 ± 6.8	80/34	4
						NRBD: 56.8 ± 10.5		
2013	Fabio *et al*.	Switzerland	NR	PSG + RBDSQ (≥6)	20	RBD: 65.5 ± 7.3	13/7	4
						NRBD: 65.8 ± 9.7		
2008	Gjerstad *et al*.	Norway	Clinical criteria (Larsen 1994); probable RBD	ICSD	245	RBD: 71.6 ± 7.9	114/117	3
						NRBD: 73.7 ± 8.5		
2014	Gong *et al*.	China	PDSBB	ICSD-2	120	66.16 ± 9.33 (44–83)	70/42	4
2015	Jankovic *et al*.	Serbia	PDSBB	ICSD-2 + RBDQ-HK	97	62.1 ± 8.8	56/41	2
2014	Kim *et al*.	Korea	PDSBB	ICSD-R	944	63.67 ± 9.33	448/496	2
2015	Kim *et al*.	Korea	NR	ICSD-R	124	62.98 ± 7.87 (34–78)	40/50	2
2009	Lee *et al*.	Korea	PDSBB	ICSD-R	447	63.8 ± 9.2	207/240	2
2016	Lim *et al*.	Korea	PDSBB	PSG + RBDSQ-K (>5)	38	RBD: 69.8 ± 6.4	20/18	2
						NRBD: 69.7 ± 7.2		
2014	Neikrug *et al*.	USA	NR	ICSD-2 + RBDSQ (≥5)	183	67.2 ± 9.4 (47–89)	43/19	4
2013	Nomura *et al*.	Japan	PDSBB; Clinical and subclinical RBD	ICSD-2	82	74.3 ± 7.2	28/31†;	4
							22/33#	
2012	Postuma *et al*.	Canada	PDSBB	ICSD-2	42	RBD: 70.5 ± 7.4	34/8	4
						NRBD: 67.5 ± 10.6		
2006	Sinforiani *et al*.	Italy	PDSBB; PD at least 5 years	ICSD	110	NRBD: 63.03 ± 8.17;	RBD-1: 42/24	3
						RBD-1§: 66.03 ± 9.19;	RBD-2: 42/33	
						RBD-2‡: 69.58 ± 7.42		
2011	Sixel-Doring *et al*.	Germany	PDSBB	ICSD-2	463	RBD: 69 ± 8	288/169	4
						NRBD: 66 ± 11		
2013	Sorensen *et al*.	Denmark	NR	ICSD	23	RBD: 62.5 ± 6.9	14/9	3
						NRBD: 60.8 ± 6.7		
2007	Vendette *et al*.	Canada	PDSBB; H-Y stage I-III	ICSD-2	34	RBD: 65.61 ± 7.73	NR	4
						NRBD: 65.13 ± 7.69		
2011	Vibha *et al*.	India	PDSBB	ICSD-R	134	RBD: 61.27 ± 8.56	90/44	2
						NRBD: 57.57 ± 11.85		
2009	Yoritaka *et al*.	Japan	Clinical criteria (Calne, 1992); H-Y stage I–IV	ICSD-R	150	68.5 ± 9.8	70/80	3
2016	Zhang *et al*.	China	PDSBB; MMSE >21	ICSD-2	46	RBD: 66.71 ± 7.21	36/10	4
						NRBD: 62.46 ± 4.8		
**Probable RBD**								
2016	Arnaldi *et al*.	Italy	Clinical criteria (Gelb 1999); de novo, drug-naïve PD	MSQ	38	RBD: 72.8 ± 6.2	24/14	3
						NRBD: 70.6 ± 7.1		
2012	Boot *et al*.	USA	Mayo Clinic Study of Aging; UPDRS ≥4	MSQ	2050	RBD: 78 (75, 82)	455/196	2
						NRBD: 77 (74, 82)		
2015	Fantini *et al*.	France	PDSBB	RBD1Q + RBDSQ (≥6)	216	RBD: 67.3 ± 9.9	130/86	3
						NRBD: 66.4 ± 11.6		
2013	Ford *et al*.	Australia	PDSBB	MSQ	124	RBD: 66.4 ± 9.9	84/40	4
						NRBD: 65.8 ± 10.9		
2015	Hu *et al*.	China	PDSBB; Probable RBD	RBDSQ (≥6)	225	RBD: 61.19 ± 11.76	117/108	3
						NRBD: 59.79 ± 10.37		
2016	Kim *et al*.	Korea	NR	RBDSQ-K (≥5)	42	62.1 ± 8.8	18/24	2
2012	Kotagal *et al*.	USA	PDSBB	MSQ (not confirmed by PSG)	80	RBD: 63.4 ± 6.7	60/20	3
						NRBD: 65.3 ± 7.1		
2014	Mahale *et al*.	India	PDSBB	RBDSQ	156	55.4 ± 11.2	119/38	2
2012	Nihei *et al*.	Japan	PDSBB	RBDSQ-J	469	71.0 ± 8.3 (44.4–91.8)	219/250	3
2005	Ozekmekci *et al*.	Turkey	NR; Movement disorders and injurious behavior during sleep	Clinical diagnostic criteria	70	RBD: 67.2 ± 8.6	54/16	2
						NRBD: 67.6 ± 9.19		
2013	Poryazova *et al*.	Switzerland	Questionnaire on sleep-wake disorders and PD characteristics	RBDSQ (≥6)	417	69 ± 9	273/131	3
2013	Suzuki *et al*.	Japan	PDSBB; Probable RBD	RBDSQ-J (≥5)	77	69.7 ± 8.9	40/37	4

**RBD**: rapid eye movement sleep behavior disorder; **NRBD**: Non-RBD; **Confirmed-RBD**: patients diagnosed according to the ICSD-2 criteria, with PSG confirmation; **Probable-RBD**: diagnosis based on interview/questionnaires, w/o PSG reconfirmation; **PDSBB**: U.K. Parkinson’s Disease Society Brain Bank criteria; **MSQ**: Mayo Sleep Questionnaire; **H-Y:** Hoehn-Yahr staging scale; **ICSD**: the International Classification of Sleep Disorders criteria for RBD; **MMSE**: Mini Mental State Examination; **RBDSQ**: the RBD screening questionnaire; **PSG**: Polysomnography.

^§^**RBD-1:** PD-RBD w/o hallucinations.

^‡^**RBD-2:** PD-RBD with hallucinations; **NR**: Not reported.

^†^Clinical RBD.

^#^Subclinical RBD.
